# A reflection on the experience of COP-3: Thailand’s perspectives

**DOI:** 10.1136/tc.2008.029256

**Published:** 2009-03-17

**Authors:** Nuntavarn Vichit-Vadakan

The COP-3 meeting in Durban holds a special memory for our team from Thailand, not only for the warm welcome we received from our gracious host and the things we accomplished but, more importantly, the connection that we discovered with those who share the same views and those who may see things differently. This COP-3 meeting brought together individuals from every corner of the world and if one could describe the meeting in one sentence, it would be that “it was vibrant”. It was “vibrant” with diverse culture and customs; “vibrant” with personalities, “vibrant” with intensity of commitment, “vibrant” with fervent discussions and “vibrant” with different views. And it was this “vibrancy” that, not only, carried us through the long and arduous days of dialogues, debates and negotiations but energised us to carry our work forward until the next meeting in 2010.

Highlights of COP-3 may differ from one party to another or from one person to the next but for many, including Thailand, it was the adoption of guidelines for article 5.3, which deals with tobacco industry interference, that made our long journey from home a success. For a variety of reasons, many anticipated even before coming to Durban that adopting guidelines for article 5.3 would be the most challenging task during COP-3. Having participated in the small working group for article 5.3, I understood the sentiments surrounding this article, which were not “either you are for the adoption or against the adoption”. Judgment made on this simplistic conclusion was counterproductive and not conducive to constructive negotiations.

What appeared to be polarisation of views among party members was merely lack of comprehension of the underlying complex issues, diverse cultural experiences and background, insecurity in making commitment owing to fear of unknown outcomes and, most importantly, a barrier in tobacco control literacy and not language proficiency. While being sensitive and respectful of others’ concerns, painstaking and intense discussions were allowed to take place during the negotiation process, which could only occur in a small group setting. As a result, views merged and trust was cultivated, which led to the more stringent guidelines for article 5.3. I would venture to state that what transpired during this small working group should be a model for future negotiations related to FCTC.

